# New Appliances & Things Medical

**Published:** 1909-02-27

**Authors:** 


### NEW APPLIANCES & THINGS MEDICAL.

[We shall be glad to receive at our Office, 28 & 29 Southampton Street, Strand, London, W.C., from the manufacturers, specimens of all new preparations and appliances.]

### GLIDINE AND IODOGLIDINE.

(Menley and James, Ltd., Farringdon Road, London, E.C., and New York.)

Although these two substances have been received by us together, they must in no sense be confused. The greater includes the less; glidine is a proteid tonic food; iodoglidine is a therapeutic agent, in which the medicinal action of iodine is favourably modified by its being in combination with glidine.

Glidine is a pure vegetable protein food, prepared from the core of a special variety of wheat peculiarly rich in proteid, and also containing an organic phosphorous compound known as lecithin. Analytical figures with which we have been provided show that this substance contains 95.69 per cent. of albumen, .87 per cent. of lecithin, .72 per cent. of ash, 2.72 per cent. carbo-hydrates. The special point about glidine is that it is entirely of vegetable origin, contains no extractive material, and that the proteid-lecithin compound is a natural and not an artificial product. The booklet which has been sent to us contains letters from a large number of Continental authorities testifying to the nutritive and remedial value of glidine in many disorders—*e.g.* diabetes, nephritis, neurasthenia, gastro-intestinal catarrh, etc. Glidine should not be heated, but it can be added either in the powder form to hot vegetables, stewed fruit, or thick soup, or it can be made into a paste with water and added in this form to any kind of food, hot or cold. It is a white, tasteless, dust-fine powder, and the usual dose is a dessert-spoonful twice or three times a day.

Iodoglidine is an organic iodine compound of which the organic radical is glidine. By its use the therapeutic effects of iodine can be obtained unaccompanied by the phenomena of iodism. It is made up in the form of tablets, each containing approximately five-sixths of a grain of iodine. The usual dose is about two tablets a day. From pharmacological reports it appears that no free iodine is split off from this compound in the stomach, and therefore no gastric irritant action is exerted by it. The action of the iodine is not cumulative; complete excretion takes place within seven days of ingestion.

### NEAVE'S HEALTH DIET.

This granular preparation of cereals in combination with the carbohydrates and proteids of milk, which has been before the medical profession and the public for some time has shown itself to be a useful addition to the equipment of the sick-room and the nursery. Recent samples indicate that the quality is maintained, and that, like the well-known Food for Infants of the same firm of manufacturers, this Health Food can be depended upon for purity and uniformity of composition. During convalescence and in old age it is usually well taken. For such conditions it is more easily digested, and less liable to induce constipation than many preparations of a similar character. The manufacturers are Messrs. Josiah R. Neave and Co., Fordingbridge, *via* Salisbury.

